# Adjusting health spending for the presence of comorbidities: an application to United States national inpatient data

**DOI:** 10.1186/s13561-017-0166-2

**Published:** 2017-08-29

**Authors:** Joseph L. Dieleman, Ranju Baral, Elizabeth Johnson, Anne Bulchis, Maxwell Birger, Anthony L. Bui, Madeline Campbell, Abigail Chapin, Rose Gabert, Hannah Hamavid, Cody Horst, Jonathan Joseph, Liya Lomsadze, Ellen Squires, Martin Tobias

**Affiliations:** 1 Institute for Health Metrics and Evaluation, 2301 5th Avenue, Suite 600, Seattle, WA 98121 USA; 2 Global Health Group, University of California at San Francisco, 550 16th Street, San Francisco, CA 94158 USA; 3 David Geffen School of Medicine at UCLA, 10833 Le Conte Ave, Los Angeles, CA 90095 USA; 4 Northwell Health, 95-25 Queens Blvd, New York, NY 11374 USA; 5 Ministry of Health, 1-3 The Terrace Level 2, Reception, Wellington, 6011 New Zealand

**Keywords:** Disease spending, Comorbidity, Comorbidity adjustment, Resource tracking, US inpatient payments

## Abstract

**Background:**

One of the major challenges in estimating health care spending spent on each cause of illness is allocating spending for a health care event to a single cause of illness in the presence of comorbidities. Comorbidities, the secondary diagnoses, are common across many causes of illness and often correlate with worse health outcomes and more expensive health care. In this study, we propose a method for measuring the average spending for each cause of illness with and without comorbidities.

**Methods:**

Our strategy for measuring cause of illness-specific spending and adjusting for the presence of comorbidities uses a regression-based framework to estimate excess spending due to comorbidities. We consider multiple causes simultaneously, allowing causes of illness to appear as either a primary diagnosis or a comorbidity. Our adjustment method distributes excess spending away from primary diagnoses (outflows), exaggerated due to the presence of comorbidities, and allocates that spending towards causes of illness that appear as comorbidities (inflows). We apply this framework for spending adjustment to the National Inpatient Survey data in the United States for years 1996-2012 to generate comorbidity-adjusted health care spending estimates for 154 causes of illness by age and sex.

**Results:**

The primary diagnoses with the greatest number of comorbidities in the NIS dataset were acute renal failure, septicemia, and endocarditis. Hypertension, diabetes, and ischemic heart disease were the most common comorbidities across all age groups. After adjusting for comorbidities, chronic kidney diseases, atrial fibrillation and flutter, and chronic obstructive pulmonary disease increased by 74.1%, 40.9%, and 21.0%, respectively, while pancreatitis, lower respiratory infections, and septicemia decreased by 21.3%, 17.2%, and 16.0%. For many diseases, comorbidity adjustments had varying effects on spending for different age groups.

**Conclusions:**

Our methodology takes a unified approach to account for excess spending caused by the presence of comorbidities. Adjusting for comorbidities provides a substantially altered, more accurate estimate of the spending attributed to specific cause of illness. Making these adjustments supports improved resource tracking, accountability, and planning for future resource allocation.

**Electronic supplementary material:**

The online version of this article (doi:10.1186/s13561-017-0166-2) contains supplementary material, which is available to authorized users.

## Background

Cause of illness spending studies aim to estimate the resources spent on preventing and treating diseases and medical conditions. One of the major challenges in measuring spending for a cause of illness is allocating spending for a health care event to a single cause of illness when comorbidities are present [1]. Although the definition of the term “comorbidity” varies slightly from study to study [2, 3], most agree that comorbidities can worsen health outcomes and increase health care spending [4–6]. Without adjusting for comorbidities in a systematic way, disease spending studies are likely to distort the true spending associated with causes of illness. This distortion stems from the high prevalence of comorbidities across many diseases and the added complexity and spending associated with being treated for multiple causes of illness at once.

Existing research assessing spending by causes of illness does not sufficiently account for comorbidities. Most studies either assign all spending to a single primary diagnosis, or divide health care spending equally across all listed diagnoses [7–10]. A few studies focus on spending for a single causes of illness and use regression-based methods to distribute spending across multiple diagnoses [4, 10–12]. While these studies do partially account for comorbidities, they tend to focus on a single disease, and risk distorting and overstating spending [1]. One systematic review of disease-specific spending studies found that the summed estimates for approximately 80 diseases added to more than double the total national health spending in the US in a single year [13].

An additional problem associated with existing comorbidity adjustment studies is that they tend to focus on chronic causes of illness [4, 10, 11]. However, the causes of illness that have the most comorbidities present are not all chronic conditions. A systematic adjustment for comorbidities that considers all major primary diagnoses and comorbidities is necessary to generate more precise estimates of cause of illness spending.

The primary diagnosis or condition is defined as the cause of illness or injury identified as the primary cause of a medical encounter. All other coexisting causes of illness or injuries are treated as secondary diagnoses, or comorbidities. Many data sources used for tracking encounter-based spending report the primary diagnosis [14]. In some routine administrative or claims-based data, the primary diagnosis in a health care event is not clearly denoted [15]. In these instances, the first diagnosis listed on a health care record is usually used as the primary diagnosis [1].

Our strategy for comorbidity adjustment uses a regression-based framework for estimating the excess spending due to comorbidities. Within this framework, we allow all causes of illness to appear as a primary diagnosis and, in most cases, also a comorbidity (of other primary diagnoses). For example, we measure the share of excess spending caused by diabetes when ischemic heart diseases is the primary diagnosis, and then also measure excess spending caused by ischemic heart disease when diabetes is the primary diagnosis. In both cases we redistribute spending away from the primary diagnosis to the comorbidity in order to generate a more accurate set of disease-specific spending estimates.

In the next section, we present our proposed method for comorbidity adjustment. This is followed by an application of our methods using data from the National Inpatient Sample. We then discuss results of the application, as well as the limitations of this research and potential future avenues for analysis.

## Methods

Our framework utilizes encounter-level data that identifies both the primary diagnosis and all comorbidities for a single health system encounter. In most cases, an encounter is a visit with a medical provider or a filled pharmaceutical prescription. A single person may have many medical system encounters in a given year. Our method redistributes resources from a primary diagnosis to the comorbidities that increase the encounter’s spending. Because a cause of illness can be a primary diagnosis in one encounter and a comorbidity in another, spending may be reallocated both from (when it is coded as primary diagnosis) and to the cause (when it is coded as comorbidity). The net change is a function of the systematic relationship between the cause of illness, other diseases, and spending patterns. We present a four-step approach for adjusting health spending data for the presence of comorbidities.

### Step1: Disease selection

To adjust for comorbidities, researchers first need to determine the primary diagnoses and the comorbidities to be included in the analysis. The set of viable comorbidities will vary based on the primary diagnosis being considered. Determining these diagnosis-comorbidity pairs is akin to defining the channels through which resources can be redistributed, and pair selection can impact results substantially.

We advocate for including as broad a set of causes of illness as possible as both primary diagnoses and comorbidities. Excluding relevant comorbidities from an analysis means that some spending, likely to be incurred because of the presence of said comorbidities, will be inappropriately attributed to the primary diagnosis. Excluding primary diagnoses leaves out some encounters entirely, and means that spending that may have resulted from the presence of a comorbidity won’t be redistributed to that comorbidity. Thus, it is critical that the set of primary diagnoses and comorbidities extends beyond a small, focused set of causes of illness.

### Step2: Modeling excess risk of spending

Next we model excess risk of spending on each primary diagnosis due to comorbidities. We estimate a separate log-linear regression for each primary condition, with spending for a health system encounter as the dependent variable and a series of binary indicators identifying the presence of each comorbidity as independent variables. The simplest form of the model is illustrated by Eq. (1):

1 $$ \ln \left({expenditure}_i\right)={\beta}_{i0}+{\sum}_{j=1}^J{\beta}_{ij}{comorbidity}_{ij}+{\varepsilon}_i $$

This equation estimates the relative risk of excess spending independently for each primary diagnosis *i* using patient or encounter level data and includes *J* comorbidities. Any other variables, such as indicators for age groups or sex, which might influence spending for a given primary condition can also be included in Eq. (1). If there is reason to believe that the excess spending due to comorbidities varies by individual characteristics, then these additional indicator variables can be interacted with the set of comorbidities. The relative risk of excess spending induced by comorbidity *j* for an index cause of illness *i* is the coefficient on the respective comorbidity (*β*_*ij*_).

When the estimated ${\widehat{\beta}}_{ij}>0$, the comorbid condition raises the spending of treating the primary diagnosis. Conversely, when ${\widehat{\beta}}_{ij}<0,$ the spending attributed to managing the primary condition is lower due to the existence of a given comorbid condition. While this is empirically rare, it would occur when a comorbid condition renders standard treatment for the primary condition ineffective, unsafe, or poorly tolerated, thus necessitating treatment that is less complex, and therefore less expensive.

### Step 3: Modelling attributable fractions

After calculating the excess risk of spending due to comorbidities, we can determine the attributable fraction (AF) of the spending on the primary diagnosis (across all encounters) that is attributable to each comorbidity. The share of total spending for primary condition *i* attributed to comorbidity *j* is the product of the relative risk of excess spending and the conditional probability of *i* and *j* occurring together. This is illustrated in Eq. (2):

2 $$ {\mathrm{AF}}_{ij}={\mathrm{p}}_{\mathrm{ij}}\left[{e}^{\widehat{\beta} ij}\hbox{--} 1\right] $$

### Step 4: Generating outflows, inflows and the comorbidity adjustment scalars

Outflows are the resources transferred away from the primary condition to comorbidities. The outflow from primary condition *i* to comorbidity *j* is the product of the attributable fraction and the total spending on the primary condition. As shown in Eq. (3), the total outflow of resources from primary condition *i* due to all comorbidities is the sum of the outflows from condition *i* to all comorbidities:

3 $$ {outflow}_i= total\ {expenditure_i}^{\ast }{\sum}_j{AF}_{ij} $$

Because a primary diagnosis for one health system encounter can be a comorbidity of another primary diagnosis for a different health system encounter, it is important that we also calculate the share of primary diagnosis *j* attributable to comorbidity *i*. If cause *i* is the diagnosis of interest, then these reverse flows can be considered inflows, or the resources transferred to cause *i* when it is reported as a comorbidity for each of the *j* other causes. As shown in Eq. (4), the total inflow of resources from all comorbidities to primary condition *i* is sum of the product of the total spending for *j* and the inflows:

4 $$ {inflow}_i={\sum}_j\ \left( total\ {expenditure_j}^{\ast }{AF}_{ij}\right) $$

The netflow of resources for a given cause of illness is the difference between total inflows and total outflows for that cause of illness, as illustrated in Eq. (5). The netflow can be positive or negative. A positive netflow means that the spending for the cause of illness increases after comorbidity adjustment, while a negative netflow means that the spending for the given cause of illness decreases after comorbidity adjustment:

5 $$ {netflow}_i={inflow}_i-{outflow}_i $$

As Eq. (6) shows, adjusted spending for cause of illness *i* is the sum of the pre-adjusted spending and the corresponding netflows:

6 $$ comorbidity adjusted\ {expenditure}_i= total\ {expenditure}_i+{netflow}_i $$

Finally, for reporting purposes or for understanding more generally how the adjustment affects each cause of illness, netflows can also be presented as comorbidity scalars by measuring the netflow as a percentage of the total spending associated with the primary diagnosis in question.

7 $$ {scalar}_i=\frac{netflow_i}{total\ {expenditure}_i}+1\kern0.75em $$

### Application

The method for comorbidity adjustment described above can be applied to any dataset with encounter-level or patient-level health spending data and multiple diagnoses recorded. We illustrate our method for comorbidity adjustment using the United States National Inpatient Sample (NIS) data for the years 1996 to 2012. The NIS is the largest publicly available, all-payer inpatient health care database in the US [16], and draws from all states participating in the Healthcare Spending and Utilization Project (HCUP). The NIS includes individuals covered by Medicare, Medicaid, or private insurance, as well as those who are uninsured. The NIS sampling frame covers more than 95% of the US population and contains the clinical and resource data included in hospital discharge records. For the purpose of this study, an encounter is defined as a hospital stay. For each encounter, the NIS includes information on multiple diagnoses and procedures, patient demographics, and total charges. NIS reports up to 24 diagnoses in addition to the primary diagnosis. Diagnoses are listed using the International Classification of Diseases version 9 (ICD-9) system [17]. The relative wealth of comorbidity information makes the NIS a rich dataset to use when studying the effect of comorbidities on health care spending. Between 1996 and 2012, the average number of observations included in the NIS was 7.6 million encounters per year.

We use the Global Burden of Disease (GBD) study 2013 as our underlying framework for disease classification [18]. In the GBD 2013 framework, ICD-9 codes are classified into 289 distinct causes based on clinically relevant groupings. Causes can be aggregated into more (or less) granular classifications depending on the policy purpose. In order to maintain enough observations per cause, we used the GBD cause level III, which classifies ICD-9 codes into 169 unique cause categories. Eight of these 169 potential causes of illness were not observed in the NIS data between 1996 and 2012.

After mapping to GBD causes, several residual cause categories remained. GBD 2013 employs E-codes, which refer to the external cause of an event such as a car crash, rather than N-codes, which refer to the nature of an event such as a broken hip. However, after mapping from ICD-9 codes to GBD causes, the data still contained N-codes for injuries. Additionally, some codes are not-elsewhere-classified (NEC), meaning that a truncated ICD-9 code caused them to be mapped to a level I or level II GBD cause, rather than level III. Probabilistic models were used to map N-codes to E-codes and to replace NEC causes with non-NEC causes in the same cause family. If a primary diagnosis was an intermediate condition rather than an underlying condition, the encounter was dropped from the sample. More details about the data cleaning process can be found in the methods Additional file 1.

Individuals were grouped into four age categories: 0 to 14 years, 15 to 44 years, 45 to 64 years, and 65 years and older. Regressions for each primary diagnosis were run independently for each age category, and all subsequent analyses were done at the age-category level.

Inpatient encounters were pooled across all years by age category and primary diagnosis. Even after pooling observations, there were several causes with relatively few observations. These were conditions that are rare in the US, such as malaria and leprosy. In order to address this issue of small numbers, causes with fewer than 1000 encounters were excluded from our analysis. The final number of causes considered for any age category in this exercise was 154. The comorbidities for each primary cause were selected based on rates of co-occurrence with the primary diagnosis. For each primary condition, comorbidities that were observed in more than 10% of the sample were considered. We excluded any intermediate causes, such as hyperlipidemia and nutrition deficiencies, as comorbidities. Residual “other” categories, such as other infections or other digestive disorders, were also excluded from analysis because these are manifestations of underlying conditions, rather than actual comorbidities.

We included binary year indicators to control for the effect of increases in spending across time and heterogeneity between sexes. Lastly, we bootstrapped the NIS data 1000 times and completed the entire analysis independently on each bootstrapped sample in order generate confidence intervals around the point estimates. All results reported are the mean estimates across all bootstrap samples.

## Results and discussion

### Presence of comorbidities

Table 1 provides summary statistics on primary diagnoses and their comorbidities for each age category. We considered a total of 47 causes for ages 0 to 14 years, 65 causes for ages 15 to 44 years, 88 causes for 45 to 64 years, and 89 causes for ages 65 years and older. The complete list of primary diagnoses and their corresponding comorbidities for each age category is found in the methods Additional file 1.

**Table 1:** Summary of primary causes of illness and comorbidities by age categories

Characteristics		0 to 14 years	15 to 44 years	45 to 64 years	65 years and older
Total number of primary diseases		47	65	88	89
Number of comorbidities observed with probabilities greater than 10%	Average	2	2	4	6
	Minimum	1	1	1	1
	Maximum	6	7	8	12
5 primary diagnoses with the greatest number of comorbidities		Acute renal failure, HIV, Cardiomyopathy and myocarditis, Protein-energy malnutrition, Drug-use disorders	Endocarditis, Septicemia, Heart failure, Acute renal failure, Aortic aneurysm	Septicemia, Acute renal failure, Endocarditis, Poisonings, Heart Failure	Endocarditis, Acute renal failure, Septicemia, Poisonings, HIV
5 causes occurring most often as comorbidities		Other infectious diseases, Congenital anomalies, Otitis media, Asthma, Endocrine, metabolic, blood and immune disorders	Well pregnancy, Tobacco, Other infectious diseases, Iron-deficiency anemia, Hypertension	Hypertension, Diabetes, Tobacco, Hyperlipidemia, Ischemic heart disease	Hypertension, Ischemic heart disease, Diabetes, Hyperlipidemia, Heart failure

The primary diagnoses that had the highest number of associated comorbidities were acute renal failure, septicemia, and endocarditis. However, there was variability in comorbidity patterns across different age groups. For example, in younger age groups, cardiomyopathy and protein-energy malnutrition had many comorbidities, while in older groups, poisonings and endocarditis had many comorbidities.

The presence of certain causes as comorbidities also varied greatly across age categories. In the youngest age category, other infectious diseases, congenital anomalies, and otitis media appeared most often as comorbidities across all primary diagnoses, with a 10.5%, 6.1%, and 5.7% probability of occurring as comorbidities, respectively. In the 15 to 44 age category, well pregnancy, tobacco, and other infectious diseases were the most common comorbidities, with a 22.8%, 13.1%, and 9.7% probability of occurring as comorbidities, respectively. In the 45 to 64 age category, hypertension, diabetes mellitus, and tobacco were the most common comorbidities across all causes, with a 39.2%, 22.1%, and 22.0% probability of occurring as comorbidities, respectively. In the oldest age group, the most common comorbidities were hypertension (47.9%), ischemic heart disease (31.0%), and diabetes mellitus (24.8%).

### Overall effects of comorbidity adjustment

**Fig. 1:**
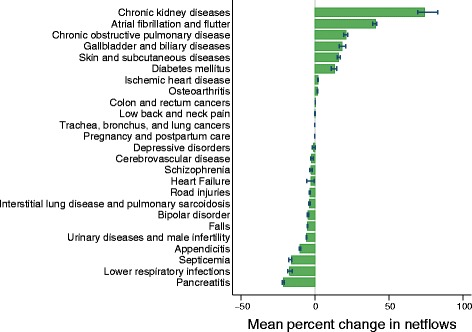
Mean percentage change in netflows for top 25 causes by spending

Figure 1 shows comorbidity scalars for the largest 25 causes of health spending reported in the NIS. Spending and scalars were pooled across all age categories.

Overall, we found that chronic kidney diseases had the highest increase in total spending after comorbidity adjustment with a 74.1% increase, followed by atrial fibrillation and flutter (40.9%). Other causes of illness with increased spending due to comorbidity adjustment were chronic obstructive pulmonary disease (21.0%), gallbladder and biliary diseases (18.2%), and skin and subcutaneous diseases (16.0%). Spending decreases due to comorbidity adjustment were most extreme for pancreatitis (21.3% decrease). Other causes with largely decreased spending included lower respiratory infections (17.2%), septicemia (16.0%), and other appendicitis (10.1%).

### Effects of comorbidity adjustment across different age groups

**Fig. 2:**
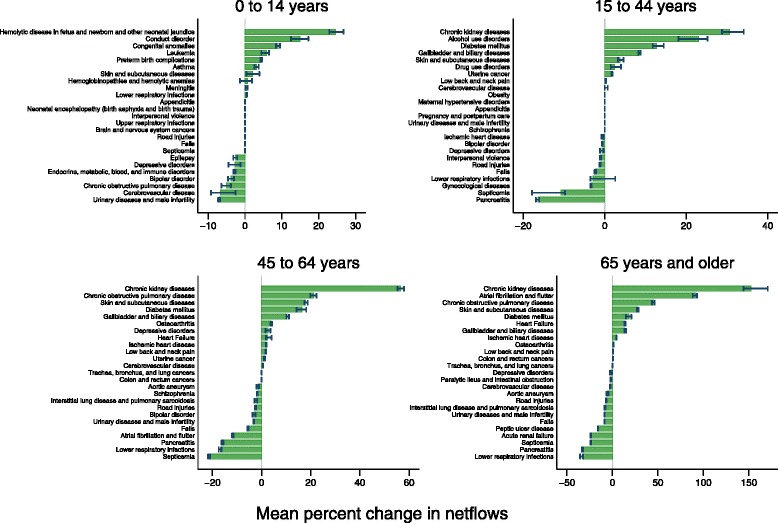
Mean percent change in netflows for top 25 causes by spending and age category

In the 0 to 14 age category, urinary diseases and male infertility, cerebrovascular disease, and chronic obstructive pulmonary disease had the largest decreases in total spending after comorbidity adjustment, with decreases of 7.0%, 6.7%, and 5.1%, respectively (Fig. 2). Bipolar disorder (3.8%), endocrine, metabolic, blood and immune disorders (2.8%), and depressive disorders (2.8%) had the next greatest decreases in spending after comorbidity adjustment. On the other hand, neonatal hemolytic disease and jaundice and conduct disorders had the largest increase in spending after comorbidity adjustment, increasing by 24.6% and 14.9%, respectively.

Among 15 to 44 year olds (Fig. 2), pancreatitis had the largest decrease in spending after comorbidity adjustment (16.5%), followed by septicemia (10.7%) and gynecological diseases (3.4%). Chronic kidney disease had the largest increase in spending after adjustment (30.6%), followed by alcohol use disorders (23.0%).

Among 45 to 64 years olds (Fig. 2), septicemia had the largest decrease in spending, with spending shrinking by 21.3%. Spending on lower respiratory infections, pancreatitis, and atrial fibrillation and flutter also decreased by more than 10.0%. The greatest increases in spending were seen in chronic kidney diseases (56.6%), chronic obstructive pulmonary disease (21.2%), and skin and subcutaneous diseases (18.0%). Similarly, spending on diabetes mellitus (16.4%) and gallbladder and biliary diseases (10.4%) increased after comorbidity adjustment.

Among those aged 65 years and older (Fig. 2), lower respiratory infections, pancreatitis, and septicemia experienced the largest decreases in spending, shrinking by 33.3%, 32.9%, and 23.8%, respectively. The greatest increases in spending were observed for chronic kidney diseases (152.5%), followed by atrial fibrillation and flutter, chronic obstructive pulmonary disease, and skin and subcutaneous diseases, with increases of 91.8%, 45.6%, and 28.0%, respectively.

### Flows of spending from primary diagnoses to comorbidities

Table 2 illustrates the flow of funds from primary diagnoses to comorbidities aggregated to GBD cause level II. Primary diagnoses are listed in the left-most column, and comorbidities are listed in the top row. The cells show the value of flows from the primary diagnoses on the left to the comorbidities across the top, with the largest flows of funds highlighted in red. The largest flow of spending, $126.3 million, is from CVD (cardiovascular disease) causes to other CVD causes. These are followed by flows from CVD to diabetes, urogenital, blood, and endocrine diseases ($79.4 million); diarrhea, lower respiratory, and other infectious diseases to chronic respiratory diseases ($66.9 million); and diarrhea, lower respiratory, and other infectious diseases to CVD ($65.7 million).

**Table 2:** Flows of funds from primary diagnoses to comorbidities

Flows from… (primary diagnoses)	Flows to… (comorbidities)									
		HIV+ Tuberculosis	Diarrhea + Lower respiratory infections + Other	Maternal	Neonatal	Other Group I	Neoplasms	Cardiovascular Disease	Chronic Respiratory	Cirrhosis
	HIV + TB	$47,883	$4,531,036	$0	$0	$0	$0	$994,880	$110,005	$211,612
	Diar + LRI + Oth	$24,485	$13,769	$0	$0	$0	$47,572	$66,720,611	$68,146,048	$0
	NTD + Malaria	$0	$25,391	$0	$0	$0	$0	$1,149,695	$109,191	$0
	Maternal	$0	$0	$8740	$0	$0	$2105	$0	$0	$0
	Neonatal	$0	$0	$0	$5,000,277	$0	$0	$0	$0	$0
	Nutr Def	$0	$0	$0	$0	$0	$658,502	$2,794,269	$937,298	$5500
	Oth Group I	$0	$191,576	$0	$0	$0	$259,838	$37,795,965	$9,966,220	$6,049,645
	CVD	$0	$0	$0	$0	$19,502	$0	$124,905,474	$58,889,842	$242,047
	Chr Resp	$0	$513,682	$0	$0	$0	$0	$47,903,538	$2,498,621	$0
	Cirrhosis	$0	$0	$0	$0	$8847	$0	$709,456	$76,266	$0
	Digestive	$0	$0	$0	$0	$0	$0	$53,073,889	$18,199,233	$3,250,228
	Neuro	$0	$0	$0	$0	$0	$0	$4,221,722	$929,515	$0
	Mental	$0	$0	$0	$0	$0	$0	$1,790,235	$1,843,204	$3,169,743
	Diab + Urog + Hem	$0	$1407	$0	$0	$2,667,774	$1,928,858	$52,219,838	$6,657,989	$69,846
	MSK	$0	$0	$0	$0	$0	$0	$4,425,751	$1,973,361	$0
	Oth NCD	$0	$0	$0	$0	$0	$0	$7,858,818	$1,590,062	$0
	Trans Inj	$0	$0	$0	$0	$0	$0	$3,967,808	$542,680	$0
	Unint Inj	$0	$0	$0	$0	$0	$0	$49,315,528	$17,244,539	$0
	Intent Inj	$0	$0	$0	$0	$0	$0	$335,798	$138,773	$0
	War + Disaster	$0	$0	$0	$0	$0	$0	$1,678,667	$242,599	$0
		Digestive	Neuro	Mental	Diab + Urog + Hem	MSK		Oth NCD	Unint Inj	Intent Inj
	HIV + TB	$0	$0	$19,761	$4,689,271	$0		$2,533,710	$0	$0
	Diar + LRI + Oth	$0	$2,674,485	$291,420	$11,133,808	$874		$444,903	$0	$0
	NTD + Malaria	$0	$0	$0	$1,024,377	$718		$0	$0	$0
	Maternal	$0	$0	$0	$28,801	$0		$0	$0	$0
	Neonatal	$0	$0	$0	$0	$0		$3,045,133	$0	$0
	Nutr Def	$2,845,725	$338,372	$91,113	$2,091,111	$2596		$347,085	$0	$0
	Oth Group I	$44,775	$17,064	$17,959	$25,486,507	$38		$40,184,865	$0	$0
	CVD	$0	$0	$69,154	$80,605,833	$0		$3,688,585	$0	$0
	Chr Resp	$0	$0	$2,391,801	$7,912,121	$0		$975	$0	$0
	Cirrhosis	$0	$0	$466,531	$554,889	$0		$226,023	$0	$0
	Digestive	$55,037,910	$0	$1,887,148	$16,338,421	$0		$1,056,559	$0	$0
	Neuro	$0	$4094	$1,079,289	$3,549,489	$1,300,158		$2,109,469	$217	$0
	Mental	$0	$2,030,830	$8,045,814	$4,405,523	$1,364,953		$0	$331,604	$4,154,669
	Diab + Urog + Hem	$0	$793,028	$219,506	$42,779,880	$10,031,234		$7,127,645	$0	$0
	MSK	$0	$0	$1,048,294	$4,214,863	$4,447,362		$8,654,772	$0	$0
	Oth NCD	$0	$1,746,922	$1,423,131	$11,365,564	$2,917,909		$215,150	$0	$0
	Trans Inj	$0	$0	$5,222,966	$42,861	$0		$0	$0	$0
	Unint Inj	$0	$1,283,171	$11,254,512	$9,092,505	$3414		$0	$0	$0
	Intent Inj	$0	$0	$1,639,392	$20,275	$0		$0	$0	$0
	War + Disaster	$0	$38,550	$693,424	$534,395	$0		$0	$0	$0

All spending flows are shown in thousands of 2014 US dollars

## Discussion

In an effort to understand the true spending on a cause of illness or injury in an inpatient setting, we applied a systematic, multi-cause adjustment to address the reality that comorbidities often increase treatment spending, and that attributing all spending to the primary diagnosis distorts health spending tracking. Unlike cause of illness-specific solutions for addressing comorbidities, this study proposes a comprehensive approach that accounts for multiple diagnoses and the effect they may have on spending for treating primary diagnoses. This flexible method can be applied to any encounter-level or patient-level data coded with spending estimates, primary diagnoses, and comorbidities.

In applying this method to NIS data, we found that comorbidities were common in all age groups, although the frequency of comorbidities was highest in the oldest age group. This is due to differences in the distribution of comorbidities across ages, as well as differences in the amount spent on treating different conditions. The direction of the adjustment also changes for a few causes across the age groups. For example, comorbidity adjustment led to a decrease in COPD (chronic obstructive pulmonary disease) spending among the youngest age category (0 to 14 years), while adjustment led to an increase in COPD spending among the oldest age category (65 years and older). This effect is not surprising, as COPD has a high probability of occurring as a comorbidity in the oldest age category, which leads to a large inflow of funds to COPD and a corresponding increase in spending. Conversely, COPD did not appear frequently as a comorbidity in the youngest age category, thereby decreasing the size of spending inflows. Rather, when COPD was present in the youngest age category, it was associated with a large number of comorbidities, generating substantial outflow and a decrease in spending.

There was a sizeable redistribution of spending across causes of illness, ranging from an increase in total spending of 74.1% for chronic kidney diseases to a decrease of 21.3% for lower respiratory infections. Across all 154 causes of illness included in this study, our method estimates a total redistribution of $1.1 trillion between 1996 and 2012. 99 of the 154 causes had resources reallocated to or from the condition.

Results from the NIS application should not be interpreted without considering the limitations of this model and the underlying data. Most importantly, the proposed method is only as good as the underlying data and diagnosis coding. Inconsistent coding or identification of primary diagnosis will make consistent estimation of the excess spending on a primary diagnosis difficult to determine. Furthermore, results are sensitive to the manner in which ICD-9 categories are aggregated together and which causes are included and excluded as primary causes and comorbidities, as in the decision to define comorbidities at a specific threshold. In terms of the model structure itself, comorbidity contributions are assumed to be additive which does not take into account the interaction between comorbidities, an effect that is well documented in the literature [19, 20]. Although we did not investigate interactions between comorbidities, our method is flexible enough to accommodate this in future iterations. A final consideration is that we perform our analysis at the encounter level rather than the person level. This precludes associating costs with health outcomes as well as incorporating comorbidity-related spending that occurs over time. However, these are not our goals. The purpose of our method is accounting spending with specific disease and to that end this method is applicable.

Despite these limitations, this application is a useful demonstration of our approach to comorbidity adjustment. We demonstrated a simplistic model, in which primary diagnoses and comorbidities differed across age categories. We controlled for the differences in cause of illness spending and comorbidity patterns across age categories by generating separate attributable fractions for each age category. This study offers a comprehensive approach to comorbidity adjustment. We apply a systematic framework to estimate both inflows and outflows of spending across all causes in an application dataset. We account not only for the outflow of spending away from a given cause, but also for the inflow of spending from other causes. This leads to a more precise and accurate estimate of overall spending on each cause of illness. While a generalized framework for addressing comorbidity expenditure in every situation is unlikely to exist, our hope is that the wider application of this approach will improve tracking of health spending, especially where comorbid expenditure was not considered before [13].

## Conclusion

A thorough understanding of US health care spending requires knowledge of how comorbidities impact spending. Disaggregating multiple conditions present in a single patient and addressed in a single health care encounter is critical. Because our methodologies address the reality that all conditions can be included as primary diagnosis, and a comorbidity, we derive net adjustments that reflect the underlying cause of health care spending. This regression-based framework disentangles excess spending due to comorbidities across conditions, creating a clearer picture of health care spending.

## Additional file

Additional file 1: Adjusting health spending for the presence of comorbidities: an application to United States national inpatient data. (PDF 332 kb)

## Abbreviations

COPD: Chronic Obstructive Pulmonary Disease
CVD: Cardiovascular Disease
HCUP: Healthcare Spending and Utilization Project
ICD: International Classification of Diseases
NEC: Not-elsewhere-classified
NIS: National Inpatient Sample
