# Supplementary material for: Adjusting health spending for the presence of comorbidities: an application to United States national inpatient data
Source: Health Econ Rev. 2017 Aug 29;7:30. doi: 10.1186/s13561-017-0166-2 (PMC5574833; doi:10.1186/s13561-017-0166-2)
Supplement: Additional file 1: — Adjusting health spending for the presence of comorbidities: an application to United States national inpatient data. (PDF 332 kb) [file 13561_2017_166_MOESM1_ESM.pdf]

METHODS ANNEX: Adjusting health spending for the presence of comorbidities: an application to United States national inpatient data

Short title: Adjusting health spending for comorbidities

Contents

Introduction
Extracting, mapping, and cleaning data
Comorbidity selection
Modeling risk of excess spending
Calculating attributable fractions
Generating flows and adjustments

Introduction

A regression-based framework was used to model the share of spending for a health system encounter that is attributable to comorbidities. In this model, spending was transferred away from an encounter's primary diagnosis and systematically redistributed across comorbidities to more accurately reflect the true cost of treating each cause.

Extracting, mapping, and cleaning data

The National Inpatient Sample survey (NIS) was used to demonstrate our method for comorbidity adjustment because it contains multiple secondary diagnoses in addition to a primary diagnosis. The unit of analysis is an encounter, which corresponds to a single inpatient hospital stay. The NIS dataset contains on average 5.3 secondary diagnoses per inpatient encounter (Table 1).

Table 1: Average number of diagnoses (primary diagnosis and comorbidities) by year in the NIS data

Year	NIS
1996	3.6
1997	3.7
1998	3.8
1999	3.8
2000	3.9
2001	4.1
2002	4.4
2003	4.6
2004	4.8
2005	5.1
2006	5.4
2007	5.8
2008	6.3
2009	6.9
2010	7.4
2011	8.0
2012	8.2

Age, sex, primary diagnosis, secondary diagnoses (comorbidities), patient weights and health spending were extracted from the NIS data for each inpatient encounter. Diagnoses are recorded using International Classification of Disease version 9 (ICD9) classification (1), (2). All diagnoses were mapped from ICD9 code to Global Burden of Disease (GBD) 2013 cause classification. Our analysis was based on level III of the GBD cause hierarchy, which classifies causes of illness and health spending across 169 causes. These causes of illness are listed in Table 2, stratified by age group. Observations which failed to map were removed. This occurred in 17% of cases, and is due to reporting error.

Table 2: Causes of illness included in analysis by age category

0-14 years	15-44 years	45-64 years	65 years and older
Tuberculosis	Tuberculosis	Tuberculosis	Tuberculosis
HIV/AIDS	HIV/AIDS	HIV/AIDS	HIV/AIDS
Diarrheal diseases	Diarrheal diseases	Diarrheal diseases	Diarrheal diseases

Intestinal infectious diseases	Intestinal infectious diseases	Intestinal infectious diseases	Intestinal infectious diseases
Lower respiratory infections	Lower respiratory infections	Lower respiratory infections	Lower respiratory infections
Upper respiratory infections	Upper respiratory infections	Upper respiratory infections	Upper respiratory infections
Otitis media	Otitis media	Otitis media	Otitis media
Meningitis	Meningitis	Meningitis	Meningitis
Encephalitis	Encephalitis	Encephalitis	Encephalitis
Whooping cough	Varicella and herpes zoster	Varicella and herpes zoster	Varicella and herpes zoster
Varicella and herpes zoster	Malaria	Malaria	Other neglected tropical diseases
Other neglected tropical diseases	Cysticercosis	Other neglected tropical diseases	Protein-energy malnutrition
Maternal hypertensive disorders	Other neglected tropical diseases	Maternal hypertensive disorders	Iron-deficiency anemia
Indirect maternal deaths	Maternal hemorrhage	Complications of abortion	Other nutritional deficiencies
Other maternal disorders	Maternal sepsis and other maternal infections	Indirect maternal deaths	Sexually transmitted diseases excluding HIV
Preterm birth complications	Maternal hypertensive disorders	Other maternal disorders	Hepatitis
Neonatal encephalopathy	Obstructed labor	Protein-energy malnutrition	Other infectious diseases
Neonatal sepsis and other neonatal infections	Complications of abortion	Iron-deficiency anemia	Septicemia
Hemolytic disease and other neonatal jaundice	Indirect maternal deaths	Other nutritional deficiencies	Esophageal cancer
Other neonatal disorders	Other maternal disorders	Sexually transmitted diseases excluding HIV	Stomach cancer
Protein-energy malnutrition	Protein-energy malnutrition	Hepatitis	Liver cancer
Iron-deficiency anemia	Iron-deficiency anemia	Other infectious diseases	Larynx cancer
Sexually transmitted diseases excluding HIV	Other nutritional deficiencies	Septicemia	Lung, bronchus, and trachea cancer
Hepatitis	Sexually transmitted diseases excluding HIV	Esophageal cancer	Breast cancer
Other infectious diseases	Hepatitis	Stomach cancer	Cervical cancer
Septicemia	Other infectious diseases	Liver cancer	Uterine cancer
Brain and nervous system cancer	Septicemia	Larynx cancer	Prostate cancer
Non-Hodgkin lymphoma	Esophageal cancer	Lung, bronchus, and trachea cancer	Colon and rectum cancer
Leukemia	Stomach cancer	Breast cancer	Mouth cancer
Other neoplasms	Liver cancer	Cervical cancer	Nasopharynx cancer
Cerebrovascular disease	Larynx cancer	Uterine cancer	Other pharynx cancer

Cardiomyopathy and myocarditis	Lung, bronchus, and trachea cancer	Prostate cancer	Gallbladder and biliary tract cancer
Other cardiovascular and circulatory diseases	Breast cancer	Colon and rectum cancer	Pancreatic cancer
Heart failure	Cervical cancer	Mouth cancer	Malignant skin melanoma
Hypertension	Uterine cancer	Nasopharynx cancer	Non-melanoma skin cancer
Chronic obstructive pulmonary disease	Prostate cancer	Other pharynx cancer	Ovarian cancer
Asthma	Colon and rectum cancer	Gallbladder and biliary tract cancer	Bladder cancer
Interstitial lung disease and pulmonary sarcoidosis	Mouth cancer	Pancreatic cancer	Brain and nervous system cancer
Other chronic respiratory diseases	Other pharynx cancer	Malignant skin melanoma	Thyroid cancer
Cirrhosis	Pancreatic cancer	Non-melanoma skin cancer	Mesothelioma
Peptic ulcer disease	Malignant skin melanoma	Ovarian cancer	Hodgkin cancer
Gastritis and duodenitis	Non-melanoma skin cancer	Bladder cancer	Non-Hodgkin lymphoma
Appendicitis	Ovarian cancer	Brain and nervous system cancer	Multiple myeloma
Paralytic ileus and intestinal obstruction	Testicular cancer	Thyroid cancer	Leukemia
Inguinal, femoral, and abdominal hernia	Bladder cancer	Mesothelioma	Other neoplasms
Inflammatory bowel disease	Brain and nervous system cancer	Hodgkin cancer	Rheumatic heart disease
Gallbladder and biliary diseases	Thyroid cancer	Non-Hodgkin lymphoma	Ischemic heart disease
Pancreatitis	Hodgkin cancer	Multiple myeloma	Cerebrovascular disease
Other digestive diseases	Non-Hodgkin lymphoma	Leukemia	Hypertensive heart disease
Epilepsy	Multiple myeloma	Other neoplasms	Cardiomyopathy and myocarditis
Migraine	Leukemia	Rheumatic heart disease	Atrial fibrillation and flutter
Other neurological disorders	Other neoplasms	Ischemic heart disease	Aortic aneurysm
Schizophrenia	Rheumatic heart disease	Cerebrovascular disease	Peripheral vascular disease
Alcohol use disorders	Ischemic heart disease	Hypertensive heart disease	Endocarditis
Drug use disorders	Cerebrovascular disease	Cardiomyopathy and myocarditis	Other cardiovascular and circulatory diseases

Depressive disorders	Hypertensive heart disease	Atrial fibrillation and flutter	Heart failure
Bipolar disorder	Cardiomyopathy and myocarditis	Aortic aneurysm	Hypertension
Anxiety disorders	Atrial fibrillation and flutter	Peripheral vascular disease	Hyperlipidemia
Eating disorders	Aortic aneurysm	Endocarditis	Chronic obstructive pulmonary disease
Autistic spectrum disorders	Peripheral vascular disease	Other cardiovascular and circulatory diseases	Pneumoconiosis
Attention-deficit/hyperactivity disorder	Endocarditis	Heart failure	Asthma
Conduct disorder	Other cardiovascular and circulatory diseases	Hypertension	Interstitial lung disease and pulmonary sarcoidosis
Other mental and substance use disorders	Heart failure	Hyperlipidemia	Other chronic respiratory diseases
Diabetes mellitus	Hypertension	Chronic obstructive pulmonary disease	Cirrhosis
Acute glomerulonephritis	Hyperlipidemia	Asthma	Peptic ulcer disease
Chronic kidney disease	Chronic obstructive pulmonary disease	Interstitial lung disease and pulmonary sarcoidosis	Gastritis and duodenitis
Urinary diseases and male infertility	Asthma	Other chronic respiratory diseases	Appendicitis
Gynecological diseases	Interstitial lung disease and pulmonary sarcoidosis	Cirrhosis	Paralytic ileus and intestinal obstruction
Hemoglobinopathies and hemolytic anemias	Other chronic respiratory diseases	Peptic ulcer disease	Inguinal, femoral, and abdominal hernia
Endocrine, metabolic, blood, and immune disorders	Cirrhosis	Gastritis and duodenitis	Inflammatory bowel disease
Acute renal failure	Peptic ulcer disease	Appendicitis	Vascular intestinal disorders
Rheumatoid arthritis	Gastritis and duodenitis	Paralytic ileus and intestinal obstruction	Gallbladder and biliary diseases
Low back and neck pain	Appendicitis	Inguinal, femoral, and abdominal hernia	Pancreatitis
Other musculoskeletal disorders	Paralytic ileus and intestinal obstruction	Inflammatory bowel disease	Other digestive diseases
Congenital anomalies	Inguinal, femoral, and abdominal hernia	Vascular intestinal disorders	Alzheimer disease and other dementias
Skin and subcutaneous diseases	Inflammatory bowel disease	Gallbladder and biliary diseases	Parkinson disease

Sense organ disorders	Vascular intestinal disorders	Pancreatitis	Epilepsy
Oral disorders	Gallbladder and biliary diseases	Other digestive diseases	Multiple sclerosis
Road injuries	Pancreatitis	Alzheimer disease and other dementias	Migraine
Other transport injuries	Other digestive diseases	Parkinson disease	Tension-type headache
Falls	Epilepsy	Epilepsy	Other neurological disorders
Drowning	Multiple sclerosis	Multiple sclerosis	Schizophrenia
Fire, heat, and hot substances	Migraine	Migraine	Alcohol use disorders
Poisonings	Tension-type headache	Tension-type headache	Drug use disorders
Exposure to mechanical forces	Other neurological disorders	Other neurological disorders	Depressive disorders
Animal contact	Schizophrenia	Schizophrenia	Bipolar disorder
Foreign body	Alcohol use disorders	Alcohol use disorders	Anxiety disorders
Other unintentional injuries	Drug use disorders	Drug use disorders	Other mental and substance use disorders
Self-harm	Depressive disorders	Depressive disorders	Diabetes mellitus
Interpersonal violence	Bipolar disorder	Bipolar disorder	Acute glomerulonephritis
Exposure to forces of nature	Anxiety disorders	Anxiety disorders	Chronic kidney disease
Collective violence and legal intervention	Eating disorders	Other mental and substance use disorders	Urinary diseases and male infertility
	Autism spectrum disorders	Diabetes mellitus	Gynecological diseases
	Attention-deficit/hyperactivity disorder	Acute glomerulonephritis	Hemoglobinopathies and hemolytic anemias
	Conduct disorder	Chronic kidney disease	Endocrine, metabolic, blood, and immune disorders
	Idiopathic intellectual disability	Urinary diseases and male infertility	Acute renal failure
	Other mental and substance use disorders	Gynecological diseases	Rheumatoid arthritis
	Diabetes mellitus	Hemoglobinopathies and hemolytic anemias	Osteoarthritis
	Acute glomerulonephritis	Endocrine, metabolic, blood, and immune disorders	Low back and neck pain
	Chronic kidney disease	Acute renal failure	Gout
	Urinary diseases and male infertility	Rheumatoid arthritis	Other musculoskeletal disorders
	Gynecological diseases	Osteoarthritis	Congenital anomalies

	Hemoglobinopathies and hemolytic anemias	Low back and neck pain	Skin and subcutaneous diseases
	Endocrine, metabolic, blood, and immune disorders	Gout	Sense organ diseases
	Acute renal failure	Other musculoskeletal disorders	Oral disorders
	Rheumatoid arthritis	Congenital anomalies	Road injuries
	Osteoarthritis	Skin and subcutaneous diseases	Other transport injuries
	Low back and neck pain	Sense organ diseases	Falls
	Gout	Oral disorders	Drowning
	Other musculoskeletal disorders	Road injuries	Fire, heat, and hot substances
	Congenital anomalies	Other transport injuries	Poisonings
	Skin and subcutaneous diseases	Falls	Exposure to mechanical forces
	Sense organ diseases	Drowning	Animal contact
	Oral disorders	Fire, heat, and hot substances	Foreign body
	Road injuries	Poisonings	Other unintentional injuries
	Other transport injuries	Exposure to mechanical forces	Self-harm
	Falls	Animal contact	Interpersonal violence
	Drowning	Foreign body	Exposure to forces of nature
	Fire, heat, and hot substances	Other unintentional injuries	Collective violence and legal intervention
	Poisonings	Self-harm	
	Exposure to mechanical forces	Interpersonal violence	
	Animal contact	Exposure to forces of nature	
	Foreign body	Collective violence and legal intervention	
	Other unintentional injuries		
	Self-harm		
	Interpersonal violence		
	Exposure to forces of nature		
	Collective violence and legal intervention		

ICD9 uses two coding systems to classify injuries: N-codes and E-codes. E-codes, which state the external cause of injury or poisoning, are most similar to the way GBD classifies injuries. However, in the NIS, E-codes are not listed as primary diagnoses. To accurately capture health system encounters resulting from injuries classified using E-codes, the E-code listed first was considered the primary diagnosis. The one exception to this rule was E-codes corresponding to adverse medical treatment. These E-codes were not allowed to be primary diagnoses because they are injuries generally occurring due to treatment complications, and are thus not typically the underlying reason for the health systems encounter. If multiple E-codes were listed for an observation, the first E-code that was not adverse medical treatment was selected as the primary diagnosis.

ICD9 codes corresponding to non-disease well person care were not allowed to be primary diagnoses for inpatient services. Similarly, encounters that violated GBD age or sex restrictions (shown in Table 2), such as females with prostate cancer or adults with neonatal illnesses, were also excluded. Finally, ICD9 codes with a primary diagnosis that was mapped to intermediate causes of illness rather than underlying causes were also removed. When a primary diagnosis was mapped to a viable cause, but secondary diagnoses were not, these secondary diagnoses were removed.

After mapping from ICD9 codes to GBD causes, the data still contained N-codes for injuries. A probabilistic replacement was used to replace these remaining N-codes with E-codes (and then mapped to GBD cause). Probability maps for this probabilistic assignment were created by pooling data across all years to make age-specific E-code probabilities, conditional on having each N-code. The conditional probabilities used in this assignment were calculated using full four- or five-digit codes from NIS.

EXAMPLE. N-code proportions and replacement

Among 55-year-olds, the GBD injury causes and the corresponding probability weights associated with the N-code N11 (Dislocation of hip) are:

- Animal contact (0.008)
- Exposure to mechanical forces (0.017)
- Other transport injuries (0.011)
- Other unintentional injuries (0.16)
- Road injuries (0.370)
- Falls (0.440)

Thus, whenever N11 appeared in the diagnosis list for 55-year-olds, it was remapped as falls in 44% of observations, as road injuries in 37% of observations, etc.

An additional challenge of mapping ICD9 codes to GBD causes occurred when abbreviated ICD9 codes (not full, 5-digit ICD9 codes) led to a diagnosis mapping to a level I or II GBD cause, rather than level III. We referred to these cases as “not elsewhere classified” (NEC). For the few NEC that existed in the NIS, a probabilistic replacement was used to replace NEC causes with viable GBD level III causes. The data were combined across all years to make age-specific probability maps. These maps were stratified by age because disease burden and the distribution of causes are a function of age. These maps were used to probabilistically reassign NEC causes to non-NEC causes.

EXAMPLE. NEC proportions and replacement

Among 55-year-olds, the causes in the cardiovascular disease (CVD) family and their corresponding probabilities of occurrence are:

- Endocarditis (0.003)
- Rheumatic heart disease (0.006)
- Cardiomyopathy and myocarditis (0.007)
- Aortic aneurysm (0.008)
- Hypertensive heart disease (0.012)
- Peripheral vascular disease (0.030)
- Atrial fibrillation and flutter (0.070)
- Other cardiovascular and circulatory diseases (0.112)
- Heart failure (0.144)
- Cerebrovascular disease (0.151)
- Ischemic heart disease (0.457)

Thus, whenever a CVD NEC cause appeared in the diagnosis list for 55-year-olds, it was remapped as ischemic heart disease in 45.7% of observations, cerebrovascular disease in 15.1% of observations, etc.

If a single observation had multiple diagnoses with the same GBD cause (for example, two or more secondary diagnoses of septicemia), the duplicate comorbidities were removed.

Encounters were divided into four age categories. The four age categories were: (1) 0-14 years, (2) 15-44 years, (3) 45-64 years, and (4) 65 years and above. These age groups are intended to roughly identify major life stages and are valuable because age groups beyond 5-year groupings have distinct patterns of comorbidity and health care utilization. While the groups are not precisely associative, other research has utilized the same age groupings. These groups have been relied on previously for reporting spending stratified by distinct life stages.[4]

We split our data by primary diagnosis and age group, pooling across sex and time. Despite pooling across these dimensions, there were several rare causes of illness, such as malaria and leprosy, with few observations. Rather than trying to estimate the effect of comorbidities on spending for these causes of illness, causes with fewer than 1,000 reported encounters across all years and both sexes within an age category were excluded from analysis.

In order to generate uncertainty intervals, 1,000 bootstrap samples were generated. All subsequent steps in comorbidity analysis were carried out 1,000 times, once for each sample. All reported results are the mean estimates across the bootstrap samples, and uncertainty is reported as the 2.5 and 97.5 percentile across the bootstrap draws.

Comorbidity selection

To be comprehensive, nearly all conditions present in the data were included in analysis. In a few cases, the comorbidities that were allowed for a primary diagnosis were restricted due to research aims and data availability.

Infrequently occurring comorbidities seemed less likely to be systematically related to price and greatly increased the computation needed for this analysis. To address this, comorbidities were excluded for a given primary diagnosis if their probability of occurring was less than 0.1. This threshold ensured that

only the most relevant and common comorbidities were included in the analysis for each primary diagnosis.

Figure 1: Distribution of comorbidities for 155 primary causes. Shown for the 65+ age group in NIS.

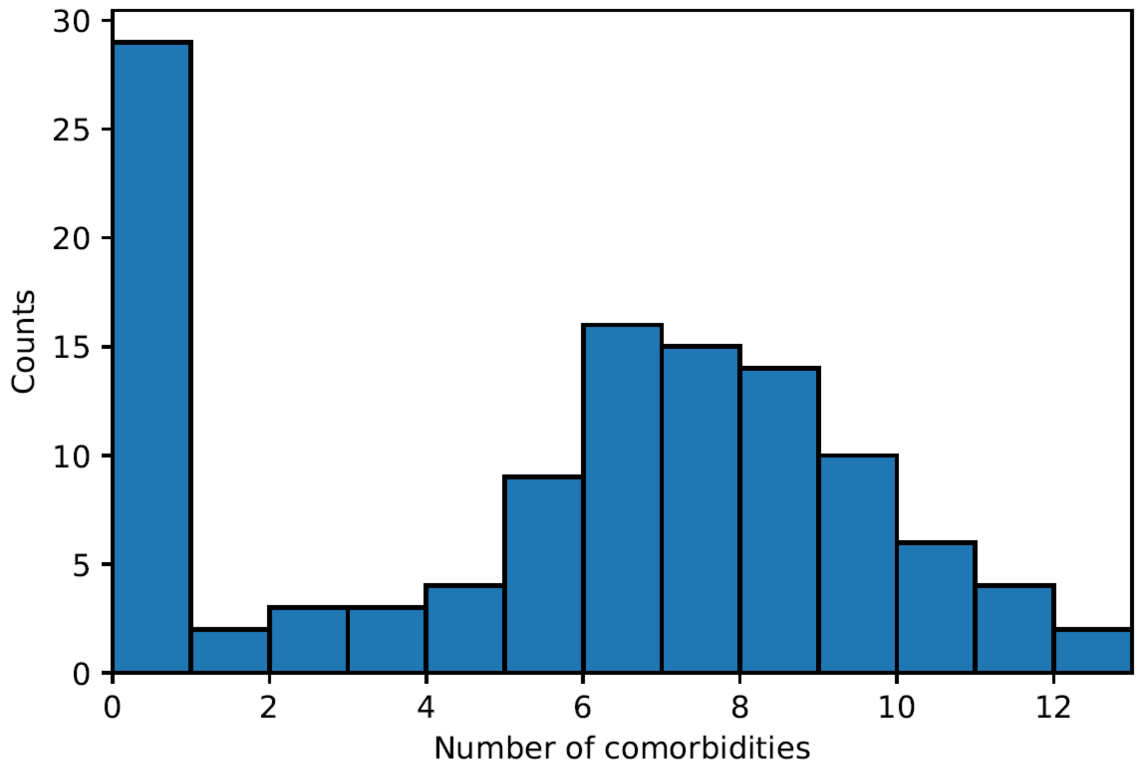

Figure 1 shows the distribution of comorbidities included for each primary diagnosis. 75% of primary diagnoses have at least one comorbidity. 68% of all primary diagnoses have at least four comorbidities.

Figure 2: Mean number of comorbidities per primary cause across threshold values. Shown for the 65+ age group.

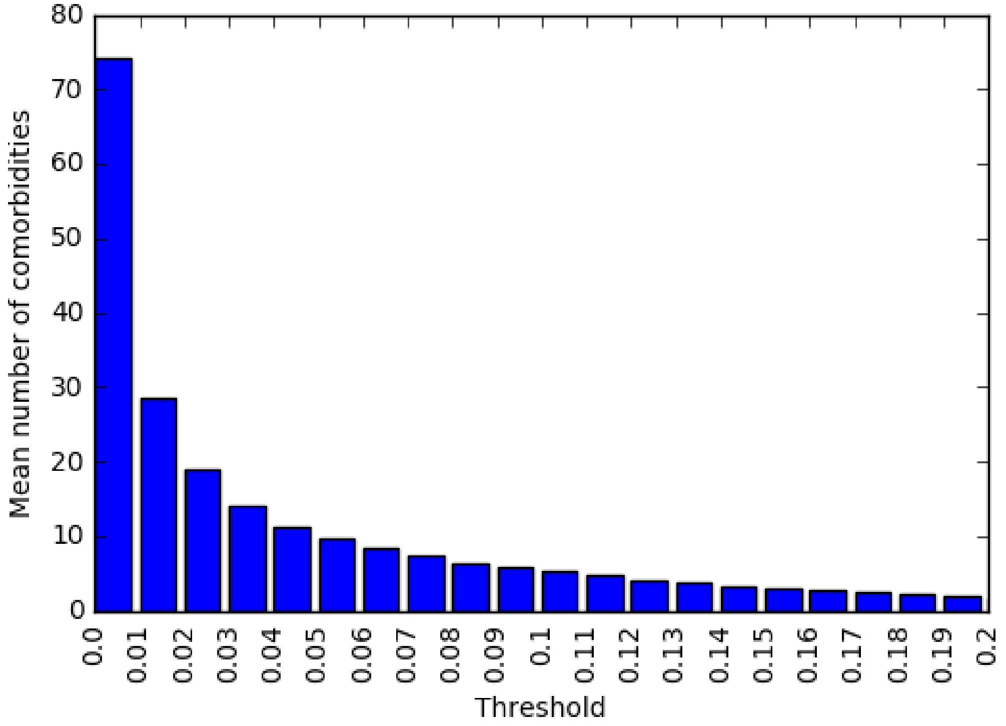

Figures 2 shows the mean number of comorbidities for different threshold values in the 65+ age group of NIS. This figure illustrates that there are a high number of low frequency comorbidities and a fairly consistent number of comorbidities once the low-frequency comorbid causes are removed.

Figure 3: Histogram of mean number of comorbidities not included per patient at a given threshold. Shown for the 65+ age group.

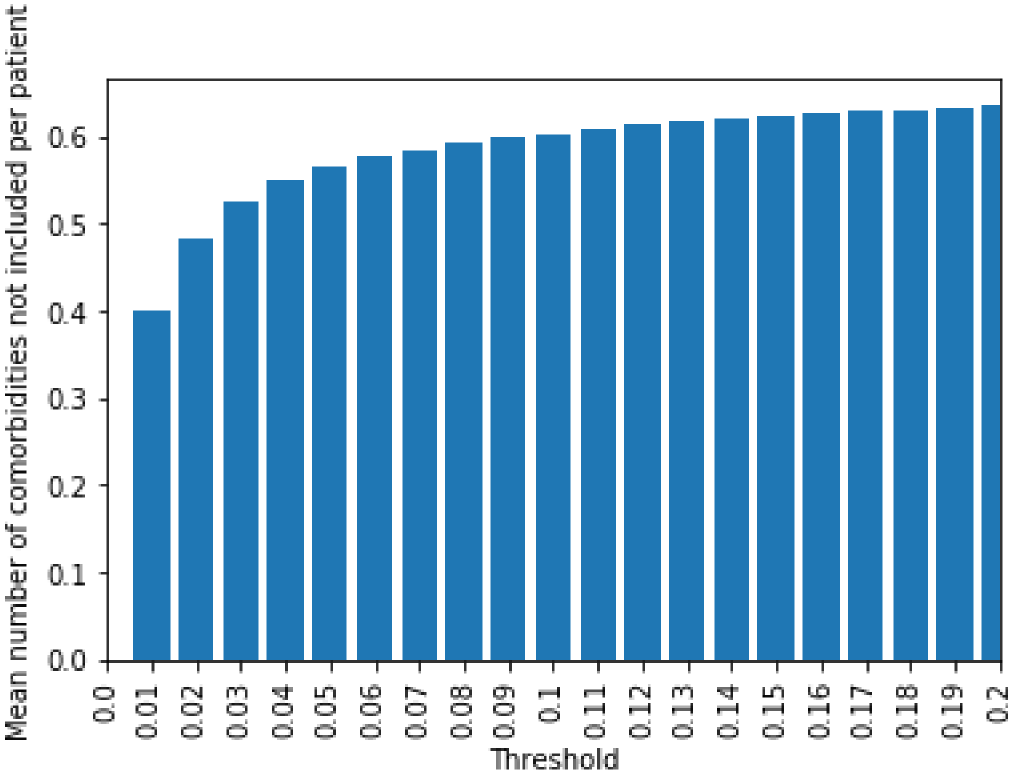

Figure 3 shows the mean number of comorbidities per person that were omitted from the analysis as a result of the frequency threshold that was applied. Using a threshold of 0.1, the average number of comorbidities excluded per patient was 0.6. The average number of comorbidities excluded per patient did not vary dramatically at different frequency thresholds.

EXAMPLE. Comorbidity pairs selection

Among 45-64 year olds, ischemic heart disease (IHD) occurs as both a primary and secondary diagnosis.

As a primary diagnosis, IHD had 145 associated secondary diagnoses. Of the 145 associated secondary diagnoses, nine had probabilities of co-occurrence greater than or equal to 0.1. Therefore, only the following secondary diagnoses were considered as comorbidities for IHD:

Comorbidity	Probability
Hypertension	0.549
Hyperlipidemia	0.514
Tobacco	0.325
Diabetes mellitus	0.316
Other cardiovascular and circulatory diseases	0.180
Heart failure	0.161
Other digestive disorders	0.127

Obesity	0.121
Chronic obstructive pulmonary disease	0.116

IHD occurred as a secondary diagnosis for 132 primary diagnoses. Of the 132 primary diagnoses, IHD occurred with a probability greater than or equal to 10% in 77. Therefore, IHD was considered as a comorbidity for 77 primary diagnoses. The top 10 primary diagnoses for which IHD was a comorbidity were:

Primary diagnosis	Probability
Heart failure	0.520
Peripheral vascular disease	0.447
Hypertensive heart disease	0.404
Rheumatic heart disease	0.402
Aortic aneurysm	0.388
Cardiomyopathy and myocarditis	0.383
Atrial fibrillation and flutter	0.345
Other cardiovascular and circulatory diseases	0.313
Chronic kidney disease	0.284
Cerebrovascular disease	0.268

We further refine the primary cause-comorbidity pairing to ensure that resources are reallocated from a primary cause only to causes that are true comorbidities, rather than manifestations of the same conditions. To do this, we apply three exclusion criteria:

1. Exclude intermediate causes. For example, remove skin and subcutaneous diseases as comorbidities when the primary diagnosis is for diabetes, and remove heart failure as a comorbidity when CVD is the primary diagnosis.
2. Exclude residual "other" categories, such as other indirect maternal causes and other infectious diseases.
3. Exclude risk factors, impairments, and well care causes, such as hyperlipidemia, renal failure, and well pregnancies.

These restrictions were set in consultation with medical professionals who have experience using ICD9 coding in clinical settings. The full list of restrictions is outlined in Table 3. Funds were not permitted to flow from the primary diagnoses in the left column to the comorbidities in the right column.

Table 3: Comorbidity restrictions

Primary diagnosis	Comorbidity
All causes	Indirect (other) maternal causes<br>Protein-energy malnutrition<br>Iron-deficiency anemia and blood related procedures<br>Other infectious diseases<br>Septicemia<br>Hypertension<br>Hyperlipidemia<br>Urinary diseases and male infertility<br>Endocrine, metabolic, blood, and immune disorders

	Acute renal failure<br>Adverse effects of medical treatment<br>Heart Failure<br>All “other” residual causes
All causes *except* lower and upper respiratory infections	Otitis media
Preterm birth complications<br>All cancers	All comorbidities
All cardiovascular diseases	Atrial fibrillation and flutter
Diabetes	Skin and subcutaneous diseases
All injuries	Skin and subcutaneous diseases<br>Sense organ diseases<br>All injuries

Modeling risk of excess spending

A log-linear regression model was used to estimate the risk of excess spending due to comorbidities. Log-linear regression is one of the most commonly used methods for modeling health care spending data (3). A model was fit separately for each primary condition and age category. Spending for a health system encounter was the dependent variable and binary indicators identifying an encounter was coded with the relevant comorbidities were the independent variables. The simplest form of the model is illustrated by Equation (1):

$$\log(expenditure_i) = \beta_{i0} + \sum_{j=1}^{J} \beta_{ij} comorbidity_{ij} + \varepsilon_i \qquad (1)$$

Using this equation, excess spending was estimated independently for each primary diagnosis *i*, using age category-specific encounter-level data. The set of *J* comorbidities was included. Binary indicators for year and sex were included to control for heterogeneous spending across the time and sex. The relative risk of excess spending for *i* induced by comorbidity *j* is the coefficient on the primary diagnosis-comorbidity pair (β_{ij}). Only statistically significant pairs ($p < 0.05$) were included in the final comorbidity adjustment.

The presence of a comorbidity generally increases health spending for a given primary diagnosis. In these cases, $\beta_{ij} > 0$, and the comorbid condition raised the cost of managing the primary condition, on average. Conversely, when $\beta_{ij} < 0$, costs of managing the primary condition decreased because of a comorbid condition. While empirically rare, this occurs if a comorbid condition makes standard treatment for the primary condition ineffective, unsafe, or poorly tolerated, necessitating less complex, and therefore less expensive, treatment.

EXAMPLE. Understanding regression results

Among 45-64 year olds, IHD appeared as comorbidity when diabetes mellitus was the primary diagnosis. In addition, diabetes was a comorbidity for IHD as a primary diagnosis. After regression, the IHD-diabetes pair had a coefficient of 0.050. The presence of diabetes as a comorbidity made IHD more expensive to treat. The diabetes-IHD pair had a coefficient of 0.006. The presence of IHD as a comorbidity made diabetes more expensive to treat, but the effect of IHD on diabetes was less than the effect of diabetes on IHD.

Calculating attributable fractions

The relative risk of excess spending due to comorbidities was then used to calculate the attributable fraction for each primary diagnosis-comorbidity pair. An attributable fraction is the proportion of disease spending on a primary diagnosis that is attributable to a specific comorbidity. The share of total spending for primary condition *i* attributable to comorbidity *j* is the product of the pair-specific relative risk of excess spending and the conditional probability of *i* and *j* co-occurring. This is illustrated by Equation (2):

$$AF_{ij} = p_{ij}\left(e^{\beta_{ij}} - 1\right) \qquad (2)$$

EXAMPLE. Calculating attributable fractions

As seen in previous examples, the IHD-diabetes pair for 45-64 year olds has a probability of occurrence of 0.318 and a regression coefficient of 0.050. The attributable fraction for this pair is as follows:

$$\boldsymbol{AF_{IHD-diabetes} = 0.318\left(e^{0.050} - 1\right)}$$

or

$$\boldsymbol{AF_{IHD-diabetes} = 0.016}$$

This means that 1.6% of spending on the treatment and prevention of IHD is attributable to diabetes.

Generating flows and adjustment scalars

The attributable fractions for all primary diagnosis-comorbidity pairs were then used to reallocate spending from primary diagnoses to comorbidities. We applied our estimates of attributable fractions to more granular, sex-specific categories disaggregated using 5-year age groups instead of the four highly aggregated age groups.

The outflows, resources transferred away from primary diagnosis *i* to comorbidity *j*, were calculated as the product of the attributable fraction AF_{ij} and the total spending on diagnosis *i*. The total outflow of resources from primary condition *i* due to all comorbidities is the sum of the outflows from *i* to all comorbidities under consideration, illustrated in Equation (4):

$$outflow_i = total\ expenditure_i * \sum_j AF_{ij} \qquad (4)$$

A primary diagnosis in one health system encounter is often a comorbidity for another primary diagnosis in a different health system encounter. Thus, it was important to calculate not only the share of primary diagnosis *i* attributable to comorbidity *j*, but also to calculate the share of primary diagnosis *j* attributable to comorbidity *i*. These funds are inflows, or the resources transferred to *i* when it is listed as a comorbidity for each of the *j* other causes. The total inflow of resources from all comorbidities to primary diagnosis *i* was calculated as sum of the product of the total spending for *j* and the attributable fractions. Equation (5) illustrates the calculation of inflows:

$$inflow_i = \sum_j (total\ expenditure_j * AF_{ij}) \qquad (5)$$

Because the comorbidity adjustment was a redistribution of resources, the total outflows across all causes in an age category should have been equal to the total inflows in that age category. That is, money flowing out of the primary diagnoses should have been the same as the money flowing to the

comorbidities. This assumption was used to verify the calculation of outflows and inflows by age category.

The netflow of resources for a primary condition captures the transfer of resources to and from that cause. That is, the netflow for cause *i* is the difference between the total inflows and total outflows for *i*, as illustrated in Equation (6). The netflow can be positive or negative. A positive netflow indicates that inflow for a cause is greater than outflow. Causes with positive netflows generally appeared often as comorbidities, and spending typically increased as a result of comorbidity adjustment. A negative netflow indicates outflow for a cause is greater than inflow. Causes with negative netflows generally appeared often as primary diagnoses and rarely as comorbidities. Spending on these causes typically decreased after comorbidity adjustment.

$$netflow_i = inflow_i - outflow_i \qquad (6)$$

The final, comorbidity adjusted spending for cause *i* was the sum of the pre-comorbidity adjusted spending for *i* and its corresponding netflow, as shown in Equation (7):

$$adjusted\ expenditure_i = total\ expenditure_i + netflow_i \qquad (7)$$

Relative increases and decreases in spending were described using comorbidity adjustment scalars. The scalar for cause *i* is defined as the netflow for *i* as a percent of the total spending on *i*. This is shown in Equation (8):

$$scalar_i = \frac{netflow_i}{total\ expenditure_i} + 1 \qquad (8)$$

The value of the scalar represented the percent change in spending for that cause. For a given cause, a scalar greater than one represented an increase in spending, while a scalar less than one represented a decrease in spending. The scalars provided a common metric for comparing comorbidity adjustments between causes and across age categories.

EXAMPLE. Calculating outflows, inflows, netflows and adjusted spending

The attributable fractions for 45-64 year olds with a primary diagnosis of IHD are:

Comorbidity	Attributable fraction
Diabetes mellitus	0.016
Chronic obstructive pulmonary disease	0.011

Total pre-comorbidity adjustment spending for all year included in this study for 45-64 year olds was $672 billion. The pair-specific outflows for each comorbidity were:

Comorbidity		Outflow
Diabetes mellitus	$672 billion * 0.016 =	$10.8 billion
Chronic obstructive pulmonary disease	$672 billion * 0.011 =	$7.4 billion

Thus, the total **outflow** from IHD to other causes was the sum of these three outflows, or approximately $18.1 billion.

The **inflow** for IHD was the sum of the outflows from the 72 diseases for which IHD was a comorbidity to IHD. The inflow to IHD was $31.6 billion.

Thus, the netflow for IHD was $31.6 billion - $18.1 billion, or $13.5 billion. The final spending for IHD among 45-64 year olds was $685.5 billion ($672 billion pre-comorbidity spending + $13.5 billion netflow), after adjusting for all comorbidities. There was a slight increase in spending on IHD in this age group after comorbidity adjustment of about 2%:

$$scalar_{IHD} = \frac{13.5}{672} + 1 = 1.02$$

Because IHD occurred frequently as a comorbidity, it had a net increase in spending due to comorbidity adjustment.

References

1. GBD 2013 Mortality and Causes of Death Collaborators. Global, regional, and national age-sex specific all-cause and cause-specific mortality for 240 causes of death, 1990-2013: a systematic analysis for the Global Burden of Disease Study 2013. The Lancet. 2014 Dec;385(9663):117–71.

2. GBD 2013 DALYs and HALE Collaborators. Global, regional, and national disability-adjusted life years (DALYS) for 306 diseases and injuries and healthy life expectancy (HALE) for 188 countries, 1990-2013: quantifying the epidemiological transition. The Lancet. 2015 Nov 28;286(10009):2145–91.

3. Willard G Manning, John Mullahy. Estimating log models: to transform or not to transform? J Health Econ. 2001;20:461–94.

4. Dieleman JL, Baral R, Birger M, Bui AL, Bulchis A, Chapin A, Hamavid H, Horst C, Johnson EK, Joseph J, Lavado R, Lomsadze L, Reynolds A, Squires E, Campbell M, DeCenso B, Dicker D, Flaxman AD, Gabert R, Highfill T, Naghavi M, Nightingale N, Templin T, Tobias MI, Vos T, Murray CJL. US Spending on Personal Health Care and Public Health, 1996-2013. JAMA. 2016 July 5;316(24):2627-2646.
